# Association between Sleep Duration and Measurable Cardiometabolic Risk Factors in Healthy Korean Women: The Fourth and Fifth Korean National Health and Nutrition Examination Surveys (KNHANES IV and V)

**DOI:** 10.1155/2016/3784210

**Published:** 2016-11-13

**Authors:** Hyeyeon Min, Yoo Jin Um, Bum Sup Jang, Doosup Shin, EunJoo Choi, Sang Min Park, Kiheon Lee

**Affiliations:** ^1^Department of Family Medicine, Seoul National University Hospital, Seoul, Republic of Korea; ^2^Department of Family Medicine, Seoul National University Bundang Hospital, Seoul National University College of Medicine, Seoul, Republic of Korea; ^3^Department of Education and Research, Seoul National University Hospital, Seoul, Republic of Korea; ^4^Department of Family Medicine, Seoul National University Hospital, Seoul National University College of Medicine, Seoul, Republic of Korea

## Abstract

*Study Objectives.* To examine the association between sleep duration and prevalence of metabolic syndrome (MetS) and its components in healthy Korean women.* Design.* Cross-sectional study, using the Fourth and Fifth Korean National Health and Nutrition Examination Surveys.* Methods.* Among 8505 women (25–70 years) from KNHANES IV and V, participants were classified into five sleep groups based on self-reported sleep duration. MetS and its components were defined using the criteria set forth in National Cholesterol Education Program-Adult Treatment Panel III. We used multivariate logistic regression analysis.* Results.* After adjusting for various confounders, shorter sleep duration (≤6 h) was found to have an association with low risk of reduced high-density lipoprotein cholesterol and increased triglycerides, whereas very long sleep duration was found to have high risk of increased triglycerides. However, abdominal obesity showed an opposite trend: short sleep duration was associated with higher risk of abdominal obesity than long sleep duration. Fasting glucose levels increased as sleep duration increased, but without significance. Moreover, blood pressure was not significantly associated with sleep duration. Consequently, MetS was less prevalent in those with short sleep duration.* Conclusions.* Sleep duration was positively associated with MetS, especially dyslipidemia and fasting hyperglycemia, but inversely associated with abdominal obesity.

## 1. Introduction

Cardiovascular disease (CVD) is a major concern not just in Korea, but also globally. Metabolic syndrome (MetS) is associated with various cardiometabolic risk factors, including central obesity, glucose dysregulation, elevated blood pressure, and dyslipidemia, resulting in higher mortality. Recently, the prevalence of MetS has sharply increased in Korea recently [1], affecting 32% of men and 26% of women, according to the Ministry of Health and Welfare of Korea in 2010 [2]. The factors contributing to the pathogenesis of MetS are poorly understood. Sleep duration has been suggested as a potential risk factor for MetS and/or its components, but the few studies that examine the impact of sleep duration on MetS report heterogeneous findings [3]. Moreover, there is a disagreement about whether the components of MetS represent a unique physiologic substructure or are merely a cluster of health hazards [4]. In addition, most previous studies with regard to the association between sleep duration and components of MetS were conducted on Caucasian populations [4–12].

Some studies indicated that sleep duration might be associated with cardiometabolic outcome in women only [6, 8, 9, 12–14]. Moreover, MetS seems to worsen the prognosis of CVD in women and is associated with a greater risk for CVD in women compared to men [4–9, 13]. In addition to this, the prevalence of sleep disturbance in women has been reported to be high [15]; however, most studies on sleep disturbances have been conducted in men [16]. There is a need to investigate this association between sleep duration and MetS in Korean women. To this end, in the current study, we used reliable data from the Fourth and Fifth Korea National Health and Nutrition Examination Surveys (KNHANES IV and V), which are cross-sectional surveys of a nationally representative Korean population. We hypothesized that both short and long sleep duration are associated with increased risk for cardiometabolic disease in Korean women.

## 2. Methods

### 2.1. Study Population

**Figure 1:**
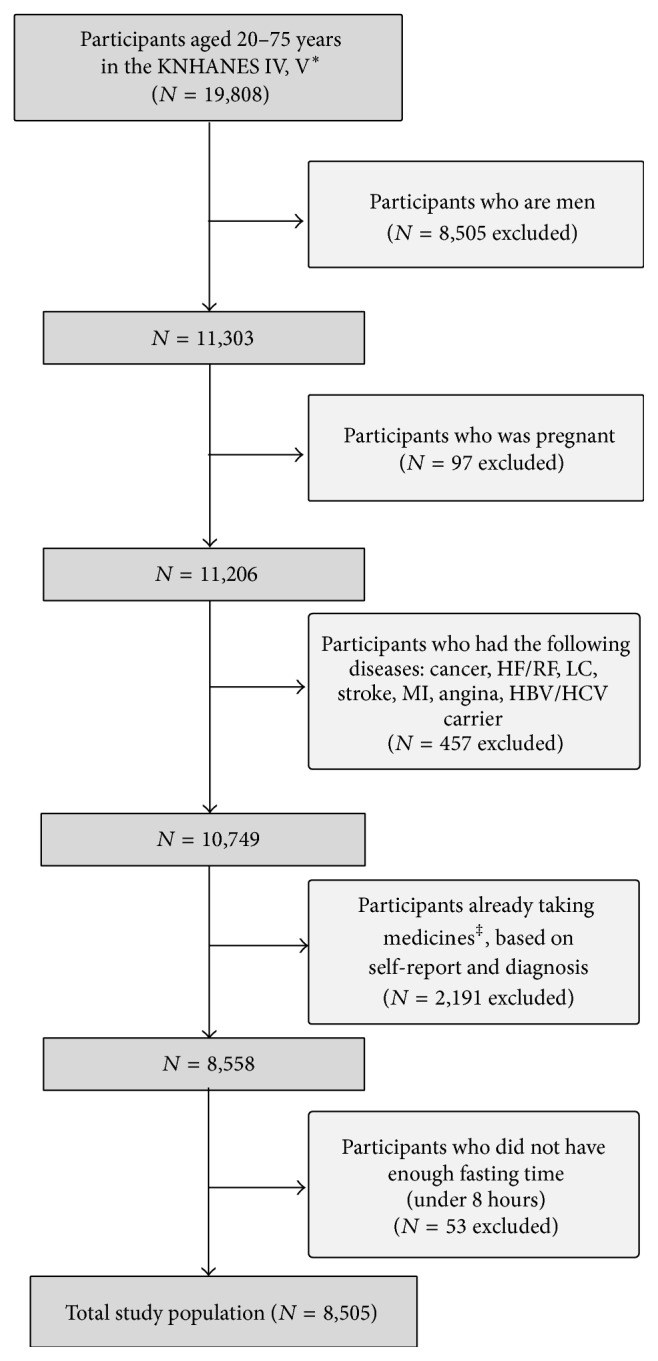
Participants flowchart. ^*∗*^The Fourth and Fifth Korean National Health and Nutrition Examination Surveys. ^†^Ordinary activities including working, studying, chores, or leisure activities. ^‡^Medicines for antihypertensive, antihyperlipidemia, antihyperglycemic effect. HF: hepatic failure; RF: renal failure; LC: liver cirrhosis; CRF: chronic renal failure; MI: myocardial infarction; HBV: hepatitis B virus; HCV: hepatitis C virus.

KNHANES IV and V (2007–2012) are nationwide surveys that represent the noninstitutionalized Korean population and are conducted by the Korea Centers for Disease Control and Prevention. It consists of the Health Interview Survey, Health Examination Study, and Nutrition Survey. A stratified, multistage probability sampling design was used, and all subjects provided informed consent prior to inclusion. Initially, there were 11,303 female adults (20–75 years) recruited from KNHANES IV and V. From this, 97 pregnant women were excluded due to physiological changes that occur during pregnancy. Moreover, we excluded 457 participants who reported that they were diagnosed with chronic renal failure, congestive heart failure, angina, myocardial ischemia, stroke, and several types of cancers (including stomach, liver, colon, breast, lung, and cervical cancer), and those who were carriers of hepatitis B or C. Participants who were on antihypertensive, antidyslipidemic, or antihyperglycemic medications were also excluded (*n* = 2.191). Lastly, participants who did not have enough fasting time (under 8 hours) before blood sampling were excluded (*n* = 53). Thereafter, the final study group was comprised of 8505 relatively healthy women, between the ages of 20 and 75 years.  Figure 1 illustrates the inclusion and exclusion process.

### 2.2. Diagnosis of MetS

Based on the criteria of the National Cholesterol Education Program-Adult Treatment Panel III (NCEP ATP III), MetS was defined as the presence of three or more of the following [17]: (1) HDL cholesterol (HDL-C) < 50 mg/dL, (2) triglycerides (TG) ≥ 150 mg/dL, (3) waist circumference (WC) > 80 cm (adopted from the International Obesity Task Force criteria for the Asian-Pacific population) [18, 19], (4) blood pressure (BP) ≥ 130/85 mm Hg, and (5) fasting plasma glucose ≥ 100 mg/dL.

### 2.3. Classification of Sleep Duration

The participants filled in a self-reported questionnaire with the question: “how many hours, on average, do you sleep a day?” Based on the answer, the subjects were classified into five groups: short sleep group (≤5 h), short sleep group (6 h), reference sleep group (7 h), long sleep group (8 h), and very long sleep group (≥9 h) [20].

### 2.4. Associated Factors

Except for body mass index (BMI), the rest of the factors were self-reported: smoking status (never, past, or current), monthly income (≤$1000, $1010 to <$3000, or ≥$3000/month), alcohol consumption (never, ≤once a month, or ≥once a week)—with high-risk drinking defined as drinking more than five cups (standard drink) on average per occasion and drinking more than two times per week. Average physical activity per week was calculated using the metabolic equivalent [21]. To determine the participants' health behavior, we additionally calculated intense physical activity per week. Intense physical activity was defined as activities that caused gasping or involved harder movements. Energy intake (kcal) was estimated using the 24-hour recall method. Education status was categorized into three levels: ≤elementary school, middle/high school, and ≥college. BMI was determined by calculating the body weight and height.

### 2.5. Anthropometric and Laboratory Measurements

Trained personnel measured the body weight and height using standard protocols to the nearest 0.1 kg and 0.1 cm, respectively. Blood pressure was manually measured using a mercury sphygmomanometer, and the values were corrected based on height of the arm during measurement, which was in accordance with the recommendation by the American Heart Association [22].

Blood samples were taken only if the participant had fasted for at least 8 h. Triglycerides (TG), HDL cholesterol, and fasting glucose were measured using ADIVIA1650 (Siemens/USA) from July 2007 to February 2008 and Hitachi Automatic Analyzer 7600 (Hitachi/Japan) from February 2008 to December 2012. Each measured HDL cholesterol value was corrected by the Lipid Standardization Program (LSP), as recommended by the Centers for Disease Control and Prevention. In each session, serum insulin was measured using the Gamma Counter (Hewlett Packard/USA) and 1470 WIZARD Gamma Counter (PerkinElmer/Finland).

### 2.6. Statistical Analysis

The characteristics of the study population were compared according to sleep duration, via an analysis of variance for continuous variables and chi-squared test for categorical variables. Logistic regression analysis was used to estimate the odds ratios for MetS and its components for each group. We chose 7 h of sleep a day as the reference category, because the average sleep duration is about 7 h a day in our study population. Model 1 was adjusted for socioeconomic variables, such as age, monthly income, and educational level. In Model 2, in addition to the adjustments made in Model 1, behavior patterns (smoking status, physical activity, alcohol intake, and energy intake) were also considered in the adjustment. Finally, BMI was additionally adjusted for in model 3. *p* values were calculated for the trends observed in a logistic regression analysis. Statistical significance was set at *p* < 0.05. All statistical analyses were performed using Stata/SE 12.1 (StataCorp, College Station, TX, USA).

## 3. Results

### 3.1. Characteristics of the Study Population

Table 1 represents the characteristics of the study population (*n* = 8,505) according to sleep duration. Women with shorter sleep duration were more likely to have lower monthly income and participate in more vigorous exercise than those with long sleep duration. Compared to the reference sleep group, the prevalence of MetS was higher in very short sleep group. With regard to the components of MetS, waist circumference (WC), systolic and diastolic blood pressure, TG, and fasting plasma glucose levels increased with decrease in sleep duration.

### 3.2. Sleep Duration and Cardiometabolic Risk Factors

Table 2 shows the odds ratios for the components of MetS according to sleep duration. After adjusting for potential confounding factors in model 3, the odds of having reduced HDL-C levels and high TG levels increased as sleep duration increased (*p*-trend < 0.01). According to model 3, subjects who slept for less than 5 h had 18% and 13% lower odds, and subjects who slept for 6 h had 14% and 19% lower odds for reduced HDL-C and high TG levels, respectively, compared to those who slept for 7 h. In addition, sleep duration of ≥9 h was associated with 1.48 times higher odds ratios for high TG levels and 1.13 times higher odds ratios for reduced HDL-C levels. Conversely, an inverse trend was observed for abdominal obesity; according to Models 1 and 2, the odds ratios for elevated WC decreased as sleep duration increased (*p*-trend < 0.01). This trend was attenuated in model 3, and thereby not significant (*p* = 0.21). Impaired fasting glucose levels only showed an increasing trend as subjects slept longer, but the increase was not significant. High blood pressure was associated with sleep duration in the unadjusted model, but the association was not significant in the multivariable-adjusted models.

### 3.3. Sleep Duration and MetS

Table 3 shows the association between sleep duration and MetS. A unique association was observed between sleep duration and prevalence of MetS in the unadjusted model. However, after adjusting for BMI (model 3), the odds ratios for MetS increased as sleep duration increased (*p* < 0.01).

Age and BMI appear to be large, independent confounders of MetS when comparing short and long sleep durations with normal sleep duration. Therefore, we analyzed more specifically the MetS according to sleep duration by age and BMI (Table 4). We categorized the participants into 4 subgroups by using age (by 40 years old) and BMI (by 25 kg/m^2^; the threshold of obesity in Korean population) cutoff points. The odds ratios of subgroups with larger BMI (over 25 kg/m^2^) increased as sleep duration increased. Next, we analyzed the association between metabolic syndrome and sleep duration via a stratification for BMI and age (Table 5). In the young-age group (≤40 years), very long sleep duration was associated with 2.00 times higher odds ratios for MetS. In the old-age group (>40 years), very short sleep duration was associated with 28% lower odds ratios for MetS. In addition, the odds ratios for MetS in both age groups increased as sleep duration increased. Meanwhile, in the higher BMI group (over 25 kg/m^2^), very long sleep duration was associated with 1.52 times higher odds ratios and very short sleep duration was associated with 38% lower odds ratios for MetS than the reference group. As noted above, the odds ratios of subgroups with higher BMI (over 25 kg/m^2^) significantly increased as sleep duration increased.

## 4. Discussion

Our results suggest that there exists an association between longer sleep duration and increased risk of MetS and its components, especially high TG, reduced HDL-C, and impaired fasting glucose levels, in healthy Korean women. Interestingly, however, an inverse trend was seen with regard to abdominal obesity.

Sleep duration has been shown to be associated with various risk factors for cardiometabolic disease [3, 4, 6, 7, 13]. Although several large epidemiologic studies have generally focused on a single cardiometabolic risk factor, the epidemiologic and clinical concepts of MetS presume that abdominal obesity, glucose dysregulation, elevated blood pressure, and dyslipidemia are a cluster of risk factors that act synergistically to influence subsequent cardiovascular diseases. Therefore, in this study, we performed a subanalysis with the components of MetS as cardiometabolic risk factors. To the best of our knowledge, this investigation is the first to evaluate the relationship between sleep duration and MetS, as well as its components in healthy Korean women.

### 4.1. Sleep Duration and Cardiometabolic Risk Factors

In this study, reduced HDL-C and high TG were associated with longer sleep duration. Since subjects with shorter sleep duration are likely to have a relatively higher socioeconomic status (educational level and monthly income) than those with longer sleep duration, they have more time to engage in healthy behaviors and seek more social support and healthcare [23]. In addition, those with shorter sleep duration are more likely to engage in physical activities (especially intense physical activities), which would naturally decrease catecholamine-induced lipolysis and TG levels and increase HDL levels [24]. However, considering that the associations were still significant after adjusting for socioeconomic factors and physical activity, we think that there may be other unknown physiologic mechanisms at play, which should be further explored in future studies. In this study, we also found that the TG levels increased significantly as sleep duration increased, which is in line with the reported epidemiologic data [25–27]. One plausible explanation for this is that long sleep duration may lead to reduced energy expenditure because more time is spent in bed. In addition, sleep quality, in addition to duration, could be a contributing factor since it has been reported that obstructive sleep apnea and sleep disturbance are more common in individuals with longer sleep duration, and these two conditions are related to insulin resistance and dyslipidemia [28]. Furthermore, previous research has suggested that people with habitually long sleep duration appear to have longer biological nights, characterized by longer periods of elevated serum melatonin and increased levels of serum cortisol, as well as longer periods with reduced body temperature [28]. Moreover, long sleep duration is likely to induce alteration of the intracellular circadian clock whereby anticipation of diurnal variations in the cellular environment, including changes in the circulating levels of nutrients (e.g., glucose, fatty acids, and triglycerides), occurs. Alterations in these molecular mechanisms, particularly within adipocytes, are likely to induce metabolic changes that may potentially increase the TG levels [25].

We found that abdominal obesity was inversely associated with sleep duration. This result is also consistent with other epidemiologic data [25, 27, 29–32]. Dysregulation of the autonomic nervous system may play a role in the association between sleep duration and obesity, which is mediated by an increase in the hypothalamic-pituitary-adrenal axis activity and the activation of systemic inflammatory processes [33]. In individuals with short sleep duration, sympathetic tone may increase and result in the accumulation of visceral fat via an inhibitory effect on the pancreatic function [34]. In particular, much attention has recently been focused on the responses of leptin, ghrelin (decreased leptin/increased ghrelin), and orexin levels on short sleep duration. Alterations in these hormone levels or patterns of secretion due to sleep deprivation may affect hunger and appetite, resulting in increased risk of overeating and consequently weight gain [9, 11, 25, 31, 35]. Furthermore, it has been reported that there exists an association between increased levels of melatonin secretion and long sleep duration; melatonin regulates inflammatory and immune processes, inhibits deposition of abdominal fat, and enhances insulin sensitivity [36].

The mechanisms underlying the association between long sleep duration and impaired fasting glucose levels are unclear. One possible mechanism is that chronic subclinical inflammation, associated with visceral obesity, may trigger long sleep duration and hyperglycemia due to the sleep-inducing and metabolic effects of proinflammatory cytokines; this may be an adaptive mechanism to promote recovery [20, 33, 37, 38]. Long sleep duration could also be reflective of sleep disorders, such as obstructive sleep apnea, which is associated with obesity, insulin resistance, and diabetes [10, 24, 28, 39].

Although some epidemiologic studies have reported a significant association between sleep duration and hypertension, other researches, including this study, could not find such association [4, 24, 40]. Since the results are conflicting and mechanisms are not clear, further studies are required to clarify the presence or absence of such association.

### 4.2. Sleep Duration and MetS

The findings from this study suggest that longer sleep duration is associated with MetS and its components, especially high TG, reduced HDL, and fasting hyperglycemia, in Korean women aged 20–75 years. Interestingly, however, an inverse association was found between longer sleep duration and abdominal obesity. From our subgroup analysis by stratification for BMI and age, sleep duration had a stronger relationship with MetS in subjects who had higher BMI (≥25 kg/m^2^). In order to interpret this finding, we further analyzed the differences of average sleep duration between the higher BMI group (≥25 kg/m^2^) and normal BMI group (<25 kg/m^2^) within each sleep duration group. As a result, in the long sleep group, obese subjects (BMI ≥ 25 kg/m^2^) tend to sleep more than normal BMI subjects (9.59 hours versus 9.47 hours, *p* = 0.06), while obese subjects in the short sleep group tend to sleep less than normal BMI subjects (5.44 hours versus 5.56 hours, *p* < 0.05). In other words, obese participants in the short sleep group may have a tendency to sleep much less compared to their counterparts, so they seem to have more negative association with MetS. Conversely, they tend to sleep more in the long sleep group; therefore, they seem to have a greater positive association with MetS. Considering that extreme sleep duration seems to be closely related to MetS, we could confidently suggest that sleep duration may have a positive association with MetS. In addition, from these findings, it seems that these components may synergistically affect the pathogenesis of cardiometabolic diseases. Further research is needed to confirm these findings and investigate the biological mechanisms that link sleep duration with MetS and its components (cardiometabolic risk factors). Additionally we analyzed the relationship between sleep duration and metabolic syndrome in Korean men population (from KNHANES IV and V) for the comparison with women population. In Korean men, sleep duration was negatively associated with metabolic syndrome, but not significant (see supplementary Table  2 in Supplementary Material available online at http://dx.doi.org/10.1155/2016/3784210).

### 4.3. Limitations and Strengths

One of the limitations of this study is the cross-sectional nature of the data, which precludes causal inferences regarding the relationship between sleep duration and MetS. Additional experimental and prospective observational studies are needed to evaluate the extent to which sleep duration affects, or is affected by, MetS and its components. We excluded some participants who take antihypertensive, antidyslipidemic, or antihyperglycemic medications because we wanted to investigate the relationship between sleep duration and metabolic syndrome only in healthy Korean women. However, such exclusion did not fully meet the ATP III criteria; hence, we additionally analyzed the relationship between sleep duration and metabolic syndrome in a population that included some participants taking antihypertensive, antidyslipidemic, or antihyperglycemic medications within the original ATP III criteria (see supplementary Table  1). Furthermore, we can find the same trend about sleep duration and metabolic syndrome. Another limitation is the use of data with self-reported sleep duration as opposed to a direct measurement of sleep duration. Although previous studies have shown good agreement between self-reported sleep duration and measured sleep duration using a polysomnography or actigraphic monitoring [41], the validity of utilizing a single question is still nonetheless questionable. However, because we incorporated national epidemiological survey data in this study, it would have been impractical to measure sleep duration several times. We also did not consider people who suffer from obstructive sleep apnea, which is known to be a causative factor for MetS and insulin resistance, insomnia, people who nap during the day, and those who work nightshifts. Finally, because this study was conducted on Koreans, the results cannot be generalized to other races or ethnic groups with different cardiometabolic risk profiles or different sleep patterns. Nevertheless, an important strength of this study is that we used data from KNHNES, which was a large-scale survey with reliable countrywide sampling. Furthermore, the relationship between sleep duration and MetS including its components was confirmed after making adjustments of various potential risk factors for MetS.

## Supplementary Material

In supplementary Table 2, we analyzed the relationship between sleep duration and metabolic syndrome in Korean men population (from KNHANES IV, V) for the comparison with women population. In Korean men, sleep duration was negatively associated with metabolic syndrome, but was not significant.

## Figures and Tables

**Table 1:** General characteristics of participants according to sleep duration.

	Sleep duration (hours a day)						
	≤5	6	7	8	≥9	Total	*p* value
*N*(*%)*	1,108 (13.0)	2,048 (24.1)	2,597 (30.5)	2,082 (24.5)	670 (7.9)	8505 (100)	
*Mean age (years)*	51.3 ± 14.6	43.8 ± 12.8	42.5 ± 12.7	41.1 ± 12.6	38.8 ± 14.3	43.3 ± 13.5	<0.01
*Education*							<0.01
≥college	181 (16.3)	631 (30.8)	887 (34.2)	734 (35.3)	198 (29.6)	2631 (30.9)	
Middle/high school	486 (43.9)	1022 (49.9)	1285 (49.5)	1027 (49.3)	339 (50.6)	4159 (48.9)	
≤elementary school	441 (39.8)	395 (19.3)	425 (16.4)	321 (15.4)	133 (19.9)	1715 (20.2)	
*Monthly income (dollars)*							<0.01
≥3,010	296 (26.7)	878 (42.9)	1107 (42.6)	873 (41.9)	241 (36.0)	3395 (40.0)	
1,010–3,000	457 (41.3)	843 (41.2)	1087 (41.9)	903 (43.4)	303 (45.2)	2866 (42.6)	
≤1,000	355 (32.0)	327 (16.0)	403 (15.5)	306 (14.7)	126 (18.8)	1517 (17.8)	
*Smoking*							<0.01
Nonsmoker	978 (88.3)	1883 (91.9)	2375 (91.5)	1872 (89.9)	562 (83.9)	7670 (90.2)	
Past-smoker	44 (4.0)	67 (3.3)	106 (4.1)	95 (4.6)	48 (7.2)	360 (4.2)	
Current smoker	86 (7.8)	98 (4.8)	116 (4.5)	115 (5.5)	60 (9.0)	475 (5.6)	
*High risk drinking*							<0.01
Never	449 (50.2)	607 (29.6)	734 (28.3)	570 (27.4)	203 (30.3)	5131 (60.3)	
≤once a month	347 (31.3)	746 (36.4)	957 (36.9)	765 (36.7)	209 (31.2)	2600 (30.6)	
≥once a week	312 (28.2)	695 (33.9)	906 (34.9)	747 (35.9)	258 (38.5)	774 (9.1)	
*Physical activity (METs/week)*	3359.8 ± 5467.4	2748.7 ± 4030.1	2600.5 ± 4058.9	2567.3 ± 4042.7	2234.9 ± 3898.6	2695.5 ± 4250.1	<0.01
*Intense physical activity (METs/week)*	1305.7 ± 4132.6	838.6 ± 2274.0	827.0 ± 2593.0	814.1 ± 2479.1	501.1 ± 1577.7	861.8 ± 2688.3	<0.01
*BMI (kg/m* ^*2*^)	23.5 ± 3.3	23.0 ± 3.3	22.8 ± 3.2	22.7 ± 3.4	22.4 ± 3.6	22.9 ± 3.3	<0.01
*24 h recall-total calories (kcal)*	1588.3 ± 635.9	1653.5 ± 662.6	1678.8 ± 580.2	1701.6 ± 636.1	1744.4 ± 669.8	1672.2 ± 629.9	<0.01
*Metabolic syndrome*							<0.01
Yes	240 (21.7)	308 (15.0)	396 (15.3)	288 (13.8)	106 (15.8)	1338 (15.7)	
No	868 (78.3)	1740 (85.0)	2201 (84.8)	1794 (86.2)	564 (84.2)	7167 (84.3)	
*HDL cholesterol (mg/dl)*	50.9 ± 11.1	51.8 ± 10.8	51.2 ± 10.5	51.1 ± 10.8	51.0 ± 10.8	51.3. ± 10.7	0.17
*Triglycerides (mg/dl)*	111.7 ± 69.0	101.0 ± 65.7	100.0 ± 69.5	100.0 ± 65.8	103.9 ± 66.6	102.1 ± 67.5	0.02
*Waist circumference (cm)*	79.1 ± 9.4	77.3 ± 9.3	76.4 ± 8.9	76.4 ± 9.3	75.7 ± 9.9	76.9 ± 9.3	0.01
*Fasting glucose (mg/dl)*	92.6 ± 15.0	91.5 ± 13.3	91.1 ± 11.2	91.6 ± 12.6	90.8 ± 11.2	91.5 ± 12.6	<0.01
*SBP (mmHg)*	115.3 ± 17.2	110.3 ± 14.7	109.7 ± 15.5	109.3 ± 14.6	108.7 ± 14.7	110.4 ± 15.4	<0.01
*DBP (mmHg)*	73.7 ± 10.0	72.0 ± 9.4	71.4 ± 9.9	71.2 ± 9.5	70.6 ± 9.4	71.7 ± 9.7	0.02

METs, metabolic equivalent; SBP, systolic blood pressure; DBP, diastolic blood pressure; HDL, high density lipoprotein; BMI, body mass index; data are mean + SD or number and percentages. *p* value is calculated from analysis of variance for continuous variables and chi-square test for categorical variables, respectively.

**Table 2:** Prevalence (%) and odds ratio (95% confidence interval) for the components of metabolic syndrome according to sleep duration.

		Sleep duration (hours a day)					
		≤5	6	7	8	≥9	*p*-trend
Reduced HDL	Prevalence (%)	571 (51.5)	956 (46.7)	1288 (49.6)	1036 (49.8)	331 (49.4)	
	Unadjusted	0.08 (0.94–1.24)	^*∗*^0.89 (0.79–1.00)	Reference	1.00 (0.90–1.13)	0.99 (0.84–1.18)	0.79
	Model 1	^*∗*^0.84 (0.73–0.98)	^*∗*^0.86 (0.76–0.96)	Reference	1.03 (0.92–1.16)	1.03 (0.87–1.23)	^*∗*^<0.01
	Model 2	^*∗*^0.84 (0.71–1.00)	^*∗*^0.88 (0.77–1.00)	Reference	1.04 (0.91–1.18)	1.07 (0.89–1.30)	^*∗*^<0.01
	Model 3	^*∗*^0.82 (0.69–0.97)	^*∗*^0.86 (0.75–0.98)	Reference	1.05 (0.92–1.20)	1.13 (0.93–1.38)	^*∗*^<0.01

High triglycerides	Prevalence (%)	238 (21.5)	292 (14.3)	388 (14.9)	307 (14.8)	120 (17.9)	
	Unadjusted	^*∗*^1.56 (1.30–1.86)	0.95 (0.80–1.12)	Reference	0.98 (0.84–1.16)	^†^1.24 (0.99–1.56)	^*∗*^0.02
	Model 1	1.03 (0.85–1.25)	0.89 (0.75–1.05)	Reference	1.04 (0.88–1.23)	^*∗*^1.35 (1.06–1.71)	^*∗*^0.04
	Model 2	0.89 (0.72–1.11)	^†^0.86 (0.75–1.00)	Reference	reference	^*∗*^1.38 (1.08–1.76)	^*∗*^<0.01
	Model 3	0.87 (0.70–1.09)	^*∗*^0.81 (0.49–0.99)	Reference	0.99 (0.82–1.20)	^*∗*^1.48 (1.13–1.93)	^*∗*^<0.01

Elevated WC	Prevalence (%)	515 (46.5)	744 (36.3)	856 (33.0)	658 (31.6)	193 (28.8)	
	Unadjusted	^*∗*^1.77 (0.53–2.04)	^*∗*^1.16 (1.03–1.31)	Reference	0.94 (0.83–1.06)	^*∗*^1.82 (0.89–0.99)	^*∗*^<0.01
	Model 1	^*∗*^1.17 (1.00–1.36)	1.07 (0.96–1.24)	Reference	0.99 (0.87–1.12)	0.89 (0.72–1.06)	^*∗*^<0.01
	Model 2	^*∗*^1.22 (1.03–1.45)	1.09 (0.95–1.25)	Reference	0.96 (0.84–1.11)	^†^0.81 (0.65–1.01)	^*∗*^<0.01
	Model 3	^†^1.24 (0.97–1.58)	1.08 (0.88–1.32)	Reference	1.09 (0.89–1.34)	0.93 (0.67–1.29)	0.21

Impaired fasting glucose	Prevalence (%)	186 (16.8)	306 (14.9)	355 (16.7)	294 (14.1)	86 (12.8)	
	Unadjusted	^*∗*^0.27 (1.05–1.55)	1.11 (0.94–1.31)	Reference	1.04 (0.88–1.23)	0.93 (0.72–1.20)	^*∗*^0.02
	Model 1	0.86 (0.70–1.05)	1.05 (0.89–1.24)	Reference	1.10 (0.83–1.30)	1.03 (0.79–1.33)	0.10
	Model 2	0.92 (0.73–1.15)	1.07 (0.94–1.37)	Reference	1.14 (0.94–1.37)	1.12 (0.85–1.49)	0.14
	Model 3	0.90 (0.72–1.14)	1.06 (0.87–1.28)	Reference	1.15 (0.95–1.39)	1.18 (0.88–1.57)	^†^0.06

High blood pressure	Prevalence (%)	228 (20.6)	275 (13.4)	337 (13.0)	247 (11.9)	82 (12.2)	
	Unadjusted	^*∗*^1.74 (1.44–2.09)	1.04 (0.88–1.24)	Reference	0.90 (0.76–1.08)	0.94 (0.72–1.21)	^*∗*^<0.01
	Model 1	0.90 (0.74–1.11)	0.74 (0.79–1.13)	Reference	0.98 (0.81–1.18)	1.05 (0.79–1.39)	0.27
	Model 2	0.91 (0.72–1.14)	0.91 (0.75–1.11)	Reference	0.97 (0.79–1.19)	1.06 (0.78–1.43)	0.29
	Model 3	0.90 (0.72–1.14)	0.90 (0.73–1.10)	Reference	0.97 (0.79–1.20)	1.09 (0.80–1.49)	0.20

Data of prevalence category are numbers and percentages.

The metabolic syndrome was defined using the National Cholesterol Education Program-Adult Treatment Panel III criteria.

Model 1, adjusted for age, education, and monthly income.

Model 2, adjusted for smoking and alcohol status, physical activity, and energy intake plus Model 1.

Model 3, adjusted for body mass index plus Model 2.

^*∗*^ *p* value < 0.05, ^†^ *p* value < 0.1.

**Table 3:** Prevalence (%) and odds ratio (95% confidence interval) for the metabolic syndrome meeting ATP III criteria according to sleep duration.

	Sleep duration (hours a day)					
	≤5	6	7	8	≥9	*p*-trend
Prevalence (%)	240 (21.7)	308 (15.0)	396 (15.3)	288 (13.8)	106 (15.8)	
Unadjusted	^*∗*^1.54 (1.29–1.84)	0.98 (0.84–1.16)	Reference	0.89 (0.76–1.05)	1.04 (0.83–1.32)	^*∗*^<0.01
Model 1	0.87 (0.72–1.05)	0.90 (0.76–1.07)	Reference	0.95 (0.80–1.12)	1.13 (0.88–1.45)	^†^0.07
Model 2	^†^0.82 (0.66–1.03)	0.91 (0.76–1.10)	Reference	0.93 (0.77–1.13)	1.15 (0.87–1.51)	^†^0.06
Model 3	^*∗*^0.75 (0.59–0.85)	^†^0.83 (0.68–1.02)	Reference	0.92 (0.74–1.14)	^†^1.31 (0.96–1.78)	^*∗*^<0.01

Data of prevalence category are numbers and percentages.

The metabolic syndrome was defined using the National Cholesterol Education Program-Adult Treatment Panel III criteria.

Model 1, adjusted for age, education, and monthly income.

Model 2, adjusted for smoking and alcohol status, physical activity, and energy intake plus Model 1.

Model 3, adjusted for body mass index plus Model 2.

^*∗*^ *p* value < 0.05, ^†^ *p* value < 0.1.

**Table 4:** Prevalence (%) and odds ratio (95% confidence interval) for the metabolic syndrome meeting ATP III criteria according to sleep duration by age and BMI.

	Sleep duration (hours a day)					
	≤5	6	7	8	≥9	*p*-trend
Age ≤ 40 and BMI < 25						
Prevalence (%)	7 (3.3)	18 (2.6)	24 (2.2)	25 (2.5)	8 (2.2)	
Unadjusted	1.55 (0.66–3.64)	1.18 (0.64–2.20)	Reference	1.15 (0.65–2.03)	1.00 (0.44–2.24)	0.54
Model 1	1.61 (0.68–3.84)	1.28 (0.68–2.38)	Reference	1.24 (0.70–2.19)	1.22 (0.54–2.78)	0.72
Model 2	1.99 (0.77–5.14)	1.22 (0.61–2.44)	Reference	0.93 (0.48–1.80)	1.44 (0.62–3.36)	0.47
Model 3	1.70 (0.64–4.53)	1.23 (0.61–2.49)	Reference	1.01 (0.51–1.97)	1.63 (0.69–3.88)	0.79

Age ≤ 40 and BMI ≥ 25						
Prevalence (%)	21 (31.3)	44 (27.3)	60 (29.7)	37 (23.7)	27 (45.8)	
Unadjusted	1.08 (0.59–1.96)	0.89 (0.56–1.41)	Reference	0.74 (0.46–1.19)	^*∗*^2.00 (1.10–3.62)	0.39
Model 1	1.09 (0.59–2.00)	0.87 (0.54–1.38)	Reference	0.75 (0.46–1.22)	^*∗*^2.31 (1.24–4.30)	0.25
Model 2	0.77 (0.37–1.57)	0.88 (0.52–1.48)	Reference	0.85 (0.50–1.44)	^*∗*^2.62 (1.30–5.29)	^*∗*^0.04
Model 3	0.82 (0.39–1.71)	0.76 (0.44–1.32)	Reference	0.89 (0.45–1.36)	^*∗*^2.71 (1.31–5.61)	^*∗*^0.04

Age > 40 and BMI < 25						
Prevalence (%)	95 (16.9)	115 (13.2)	121 (13.0)	100 (15.0)	27 (16.1)	
Unadjusted	^*∗*^1.36 (1.02–1.82)	1.02 (0.77–1.34)	Reference	1.18 (0.89–1.57)	1.28 (0.81–2.02)	0.70
Model 1	0.90 (0.66–1.22)	1.03 (0.77–1.36)	Reference	1.16 (0.87–1.57)	1.03 (0.64–1.66)	0.20
Model 2	0.84 (0.59–1.20)	1.07 (0.79–1.46)	Reference	1.21 (0.87–1.68)	0.94 (0.55–1.61)	0.20
Model 3	0.88 (0.61–1.27)	1.05 (0.76–1.45)	Reference	1.25 (0.89–1.75)	1.16 (0.66–2.02)	0.12

Age > 40 and BMI ≥ 25						
Prevalence (%)	117 (43.5)	131 (41.5)	191 (52.5)	126 (40.3)	44 (58.7)	
Unadjusted	^*∗*^0.70 (0.51–0.96)	^*∗*^0.65 (0.47–0.87)	Reference	0.85 (0.61–1.16)	1.29 (0.78–2.13)	^*∗*^<0.01
Model 1	^*∗*^0.62 (0.44–0.85)	^*∗*^0.65 (0.47–0.88)	Reference	0.85 (0.62–1.17)	1.16 (0.69–1.93)	^*∗*^<0.01
Model 2	^*∗*^0.60 (0.42–0.87)	^*∗*^0.68 (0.48–0.96)	Reference	0.86 (0.60–1.24)	1.12 (0.63–1.99)	^*∗*^<0.01
Model 3	^*∗*^0.59 (0.40–0.86)	^*∗*^0.66 (0.43–0.93)	Reference	0.77 (0.53–1.12)	1.08 (0.60–1.92)	^*∗*^0.01

Data of prevalence category are numbers and percentages.

The metabolic syndrome was defined using the National Cholesterol Education Program-Adult Treatment Panel III criteria.

Model 1, adjusted for age, education, and monthly income.

Model 2, adjusted for smoking and alcohol status, physical activity, and energy intake plus Model 1.

Model 3, adjusted for body mass index plus Model 2.

^*∗*^ *p* value < 0.05.

**Table 5:** Prevalence (%) and odds ratio (95% confidence interval) for the metabolic syndrome meeting ATP III criteria according to sleep duration by age and by BMI.

	Sleep duration (hours a day)					
	≤5	6	7	8	≥9	*p*-trend
Age ≤ 40						
Prevalence (%)	28 (10.1)	62 (7.2)	84 (6.5)	62 (5.4)	35 (8.2)	
Unadjusted	^*∗*^1.63 (1.04–2.55)	1.13 (0.80–1.58)	Reference	0.82 (0.59–1.15)	1.29 (0.86–2.00)	0.13
Model 1	^†^1.16 (0.82–1.63)	1.16 (0.82–1.63)	Reference	0.85 (0.601.20)	^†^1.42 (0.93–2.17)	0.28
Model 2	1.40 (0.82–2.39)	1.14 (0.79–1.67)	Reference	0.81 (0.55–1.18)	^†^1.53 (0.97–2.41)	0.57
Model 3	1.09 (0.60–1.99)	0.83 (0.53–1.29)	Reference	0.81 (0.52–1.24)	^*∗*^2.00 (1.19–3.38)	0.16
Age > 40						
Prevalence (%)	212 (25.5)	246 (20.7)	312 (24.1)	226 (24.4)	71 (29.2)	
Unadjusted	1.08 (0.88–1.32)	^*∗*^0.82 (0.68–1.00)	Reference	1.02 (0.83–1.24)	^†^1.30 (0.96–1.76)	0.23
Model 1	^†^0.81 (0.66–1.00)	^*∗*^0.82 (0.68–1.00)	Reference	1.01 (0.82–0.23)	1.09 (0.79–1.49)	^*∗*^<0.01
Model 2	^*∗*^0.79 (0.62–0.99)	0.84 (0.68–1.04)	Reference	1.01 (0.81–1.27)	1.04 (0.74–1.48)	^*∗*^0.01
Model 3	^*∗*^0.72 (0.56–0.94)	^†^0.82 (0.65–1.04)	Reference	0.97 (0.76–1.24)	1.08 (0.73–1.59)	^*∗*^<0.01

BMI < 25						
Prevalence (%)	102 (13.2)	133 (8.5)	145 (7.1)	125 (7.5)	35 (6.5)	
Unadjusted	^*∗*^1.98 (1.51–2.59)	1.20 (0.94–1.54)	Reference	1.06 (0.82–1.35)	0.91 (0.62–1.33)	^*∗*^<0.01
Model 1	0.93 (0.69–1.24)	1.08 (0.83–1.39)	Reference	1.16 (0.89–1.51)	1.02 (0.68–1.53)	0.35
Model 2	0.88 (0.63–1.22)	1.10 (0.82–1.46)	Reference	1.12 (0.83–1.50)	1.01 (0.64–1.58)	0.42
Model 3	0.93 (0.66–1.31)	1.07 (0.79–1.43)	Reference	1.18 (0.87–1.59)	1.25 (0.79–1.99)	0.19
BMI ≥ 25						
Prevalence (%)	138 (47.1)	175 (36.7)	251 (44.4)	163 (39.1)	71 (53.0)	
Unadjusted	0.87 (0.67–1.15)	^*∗*^0.73 (0.57–0.93)	Reference	^†^0.81 (0.62–1.04)	^†^1.41 (0.97–2.07)	^†^0.07
Model 1	^*∗*^0.48 (0.51–0.91)	^*∗*^0.71 (0.55–0.92)	Reference	0.82 (0.63–1.07)	^†^1.45 (0.98–2.14)	^*∗*^<0.01
Model 2	^*∗*^0.64 (0.46–0.88)	^†^0.75 (0.57–1.00)	Reference	0.86 (0.64–1.15)	^*∗*^1.17 (1.01–2.44)	^*∗*^<0.01
Model 3	^*∗*^0.62 (0.45–0.87)	^*∗*^0.70 (0.53–0.94)	Reference	0.78 (0.58–1.06)	^†^1.52 (0.97–2.38)	^*∗*^<0.01

Data of prevalence category are numbers and percentages.

The metabolic syndrome was defined using the National Cholesterol Education Program-Adult Treatment Panel III criteria.

Model 1, adjusted for age, education, and monthly income.

Model 2, adjusted for smoking and alcohol status, physical activity, and energy intake plus Model 1.

Model 3, adjusted for body mass index plus Model 2.

^*∗*^ *p* value < 0.05, ^†^ *p* value < 0.1.
