# Supplementary material for: Association between Sleep Duration and Measurable Cardiometabolic Risk Factors in Healthy Korean Women: The Fourth and Fifth Korean National Health and Nutrition Examination Surveys (KNHANES IV and V)
Source: Int J Endocrinol. 2016 Nov 13;2016:3784210. doi: 10.1155/2016/3784210 (PMC5124459; doi:10.1155/2016/3784210)
Supplement: Supplementary file 1 — In supplementary Table 2, we analyzed the relationship between sleep duration and metabolic syndrome in Korean men population (from KNHANES IV, V) for the comparison with women population. In Korean men, sleep duration was negatively associated with metabolic syndrome, but was not significant. [file 3784210.f1.docx]

Supplementary Table 1. Prevalence (%) and odds ratio (95% Confidence interval) for the Metabolic syndrome meeting ATP III criteria (included antihypertensive, antidyslipidemic, or antihyperglycemic medications) according to sleep duration

|  | **Sleep duration(hours a day)** | | | | | |
| --- | --- | --- | --- | --- | --- | --- |
|  | **≤5** | **6** | **7** | **8** | **≥9** | **p-trend** |
| **Prevalence (%)** | 241(21.7) | 309(15.1) | 397(15.3) | 292(14.0) | 107(15.9) |  |
| **Unadjusted** | *1.53(1.28-1.83) | 0.98(0.84-1.16) | reference | 0.90(0.77-1.06) | 1.05(0.83-1.33) | *<0.01 |
| **Model1** | 0.86(0.71-1.05) | 0.90(0.76-1.07) | reference | 0.95(0.80-1.13) | 1.14(0.89-1.46) | †0.06 |
| **Model2** | †0.82(0.66-1.02) | 0.91(0.76-1.10) | reference | 0.94(0.78-1.14) | 1.16(0.88-1.52) | *0.05 |
| **Model3** | *0.75(0.59-0.95) | †0.83(0.68-1.02) | reference | 0.92(0.75-1.14) | †1.32(0.97-1.80) | *<0.01 |

Data of Prevalence category are numbers and percentages.

The Metabolic syndrome were defined using the National Cholesterol Education Program-Adult Treatment Panel III criteria

Model 1, adjusted for age, education, monthly income.

Model 2, adjusted for smoking and alcohol status, physical activity and energy intake plus Model 1.

Model 3, adjusted for Body mass index plus Model 2

*p-value<0.05, †p-value<0.1,

Supplementary Table 2. Prevalence (%) and odds ratio (95% Confidence interval) for the Metabolic syndrome meeting ATP III criteria according to sleep duration in Korean men

|  | **Sleep duration(hours a day)** | | | | | |
| --- | --- | --- | --- | --- | --- | --- |
|  | **≤5** | **6** | **7** | **8** | **≥9** | **p-trend** |
| **Prevalence (%)** | 161(22.8) | 383(21.2) | 422(21.7) | 279(19.8) | 84(19.2) |  |
| **Unadjusted** | 1.07(0.87-1.31) | 0.97(0.83-1.14) | reference | 0.89(0.75-1.06) | 0.86(0.66-1.11) | 0.08 |
| **Model1** | 1.00(0.81-1.24) | 0.97(0.83-1.13) | reference | 0.89(0.75-1.05) | 0.83(0.64-1.09) | 0.16 |
| **Model2** | 0.92(0.72-1.19) | 0.94(0.78-1.12) | reference | 0.85(0.70-1.04) | 0.79(0.58-1.08) | 0.29 |
| **Model3** | 0.86(0.65-1.14) | 0.86(0.70-1.05) | reference | 0.92(0.73-1.15) | 0.84(0.59-1.19) | 0.64 |
